# Profiling of five urinary exosomal miRNAs for the differential diagnosis of patients with diabetic kidney disease and focal segmental glomerulosclerosis

**DOI:** 10.1371/journal.pone.0312470

**Published:** 2024-10-29

**Authors:** Sinan Trabulus, Mehmet Seyit Zor, Selma Alagoz, Mevlut Tamer Dincer, Meral Meşe, Erkan Yilmaz, Eda Tahir Turanli, Nurhan Seyahi

**Affiliations:** 1 Division of Nephrology, Department of Internal Medicine, Cerrahpasa Medical Faculty, Istanbul University-Cerrahpasa, Istanbul, Turkey; 2 Department of Molecular Biology and Genetics, Faculty of Science and Letters, Istanbul Technical University, Istanbul, Turkey; 3 Genome Biology Unit, European Molecular Biology Laboratory (EMBL), Heidelberg, Germany; 4 Division of Nephrology, Bagcilar Training and Research Hospital, University of Health Sciences, Istanbul, Turkey; 5 Division of Nephrology, Kartal Lutfi Kirdar Training and Research Hospital, University of Health Sciences, Istanbul, Turkey; 6 Tissue Typing Laboratory, Cerrahpasa Medical Faculty, Istanbul University-Cerrahpasa, Istanbul, Turkey; 7 Department of Molecular Biology and Genetics, Faculty of Engineering and Natural Sciences, Acibadem University, Istanbul, Turkey; 8 Program of Molecular Biology and Genetics, Institute of Natural Sciences, Acibadem University, Istanbul, Turkey; Universidade de Sao Paulo, BRAZIL

## Abstract

**Objective:**

The objective of this study is to investigate the diagnostic utility of microRNAs (miRNAs) for distinguishing between urine samples from patients with Diabetic Kidney Disease (DKD) and those with Focal Segmental Glomerulosclerosis (FSGS).

**Methods:**

In this multicentric, cross-sectional investigation, we enrolled patients diagnosed with DKD, individuals with primary biopsy-proven FSGS, and healthy controls. The top 5 miRNAs (hsa-mir-21, hsa-mir-30a, hsa-mir-193a, hsa-mir-196a, hsa-mir-200a) were selected to quantify miRNAs in urine samples. Isolation of targeted miRNAs was performed from urinary exosomes, and the quantitative profile of the isolated miRNAs was measured by RT-qPCR. The ΔΔCt method was implemented to calculate the fold differences between disease and control samples.

**Results:**

Thirteen DKD patients, 11 FSGS patients, and 14 healthy controls were included in this study. Hsa-mir-21 and hsa-mir-30a exhibited distinct regulation in both groups, with upregulation observed in FSGS and downregulation in DKD (hsa-mir-21 in DKD (0.668 ± 0.25, p < 0.0005) and FSGS (2.267 ± 1.138, p < 0.0077); hsa-mir-30a in DKD (0.874 ± 0.254, p = 0.079) and FSGS (1.378 ± 0.312, p < 0.0006)). Hsa-mir-193a exhibited significant dysregulation in DKD (1.017 ± 0.413, p < 0.029) but not in FSGS (4.18 ± 1.528, p = 0.058). Hsa-mir-196a and hsa-mir-200a showed upregulation in patient groups (hsa-mir-196a in DKD (1.278 ± 0.527, p = 0.074) and FSGS (2.47 ± 0.911, p < 0.0003); hsa-mir-200a in DKD (1.909 ± 0.825, p = 0.082) and FSGS (1.301 ± 0.358, p < 0.008)).

**Conclusion:**

Specific miRNAs, particularly miR-21, miR-30a, miR-196a, and miR-200a, might play a role in the pathogenesis of kidney diseases and could potentially serve as biomarkers to distinguish between FSGS and DKD patients.

## Introduction

Nephrotic syndrome characterized by the hyperexcretion of proteins in the urine, signifies a perturbation in the renal perm-selectivity barrier. This syndrome involves the intricate glomerular filtration barrier, comprising the fenestrated endothelium, the glomerular basement membrane, and the podocytes. Normally, this apparatus selectively filters blood plasma, excluding macromolecules exceeding 69 kD [1, 2]. Pathological destruction of podocytes beyond a critical limit precipitates irreversible glomerular damage, marking a severe progression of the disease [3].

Recently, microRNAs (miRNAs) have emerged as key biomolecular species in nephrology. All cells release small extracellular vesicles containing miRNA as part of their molecular content. Encapsulation into exosomes protects them from degradation, so the stability of this molecular cargo extends to bodily fluids like blood, urine, and saliva. The stability and specificity classify exosomal miRNAs as one of the best kinds of biomarkers [4]. Their intricate regulatory roles in both physiological and pathological contexts of renal function have garnered substantial research interest. Understanding miRNA-mediated mechanisms opens avenues for their application as biomarkers in renal pathologies [5].

Diabetic Kidney Disease (DKD) and Focal Segmental Glomerulosclerosis (FSGS) represent two primary manifestations of nephrotic syndrome. DKD, a leading cause of end-stage renal disease globally, manifests in 25–40% of individuals with diabetes mellitus [6]. miRNAs, notably miR-193a-3p and miR-21, have been implicated in the pathophysiology of DKD, influencing key factors such as glomerular filtration and proteinuria [7, 8]. FSGS, often concomitant with nephrotic syndrome, is identifiable through histopathological changes in renal biopsies. This condition is attributed to dysregulation in key podocyte-associated miRNAs, particularly those within the miR-200 family, which are crucial for podocyte structural integrity and function [9, 10].

Chronic Kidney Disease (CKD) is classified into stages based on the glomerular filtration rate (GFR), with the KDIGO 2012 clinical practice guideline for the evaluation and management of CKD. According to KDIGO, CKD stages range from G1 (normal or high GFR ≥90 mL/min/1.73 m^2^) to G5 (kidney failure, GFR <15 mL/min/1.73 m^2^ or on dialysis) [11]. Patients in stages G1 to G3b (GFR 30–89 mL/min/1.73 m^2^) typically exhibit mild to moderate kidney damage and are often asymptomatic, while stages G4 and G5 indicate severe impairment with significant clinical manifestations. Our study specifically includes participants with an estimated glomerular filtration rate (eGFR) ≥30 mL/min/1.73 m^2^, corresponding to CKD stages G1 to G3b. This criterion ensures the exclusion of severe CKD stages (G4 and G5), where advanced kidney damage could confound the interpretation of miRNA expression patterns. By focusing on patients within these stages, we aim to discern the miRNA profiles associated with early to moderate renal impairment, providing insights into their potential roles as biomarkers for DKD and FSGS before the onset of advanced kidney failure.

In our study, we aim to conduct a quantitative analysis of five critical miRNAs (miR-21, miR-30a, miR-193a, miR-196a, miR-200a) in urine samples from patients diagnosed with DKD and FSGS. We selected these miRNAs through a rigorous selection process, beginning with a search in the Human microRNA Disease Database (HMDD) for miRNAs associated with nephropathy, diabetic nephropathy, and focal segmental glomerulosclerosis, identifying 72 miRNAs. A PubMed literature review narrowed this list to 15 miRNAs. Using mirPath v3, we identified 64 pathways involving these miRNAs, focusing on key pathways integral to renal disease progression, such as TGF-beta, mTOR, and Wnt signaling. This process refined our selection to 12 miRNAs, from which we chose the top five for their significant roles in these critical pathways.

The study is designed to elucidate the differential expression patterns of these miRNAs in the context of these two distinct nephrotic syndromes. By doing so, we aspire to not only reinforce the understanding of miRNA involvement in nephrotic syndrome pathology but also to evaluate the potential of these miRNAs as specific biomarkers for distinguishing between DKD and FSGS.

## Materials and methods

### Study group

In this multicentric, cross-sectional investigation, we enrolled patients diagnosed with DKD stemming from type 2 diabetes mellitus, individuals with primary biopsy-proven FSGS, and healthy controls from hospital staff between January 3, 2018, and February 3, 2021. The study encompassed male and female participants, aged ≥40 and ≤65 years, exhibiting proteinuria >300 mg/day and an estimated glomerular filtration rate (eGFR)≥30 mL/min/1.73 m^2^. Exclusion criteria encompassed patients with type 1 diabetes mellitus, secondary FSGS, autoimmune disorders, inflammatory bowel diseases, significant hematuria (defined as >5 erythrocytes per high-power field in urine sediment in two consecutive tests), clinical or laboratory indicators of infection, malignancy, and those undergoing immunosuppressive therapy in the preceding six months.

This study was approved by the research ethics committee of Istanbul University-Cerrahpasa, Cerrahpasa Faculty of Medicine (Approval number: 457404) and certify that the study was performed in accordance with the ethical standards as laid down in the 1964 Declaration of Helsinki and its later amendments or comparable ethical standards. Written informed consent was obtained from all individual participants included in the study.

### Data collection and definitions

We compiled demographic, clinical, and laboratory data from patients’ medical records and the hospital’s electronic database. This data included age, gender, duration since diagnosis, serum creatinine levels, and 24-hour urinary albumin and protein content. Serum creatinine levels were measured using a colorimetric method with an autoanalyzer, while urine albumin and protein levels were measured using an immunoturbidimetric method with an nautoanalyzer.

The eGFR was computed utilizing the Chronic Kidney Disease Epidemiology Collaboration (CKD-EPI) 2009 formula [12]. Albuminuria and proteinuria quantifications were conducted on 24-hour urine collections.

Diabetes Mellitus diagnosis adhered to the Standards of Medical Care in Diabetes 2021 criteria [13]. DKD was characterized by the presence of albuminuria (>30 mg/24 h) in type 2 diabetes patients. FSGS diagnosis was based on histopathologic findings including segmental sclerosis, glomerular adhesion to Bowman’s capsule, and damage to renal podocytes. Secondary FSGS, attributed to infections, drugs, or other conditions, was excluded.

### miRNA discovery

HMDD provides curated disease-miRNA associations with experimentally supported evidence [14]. Initially, we searched HMDD using keywords such as ’Nephropathy’, ’Diabetic Nephropathy’, and ’Focal Segmental Glomerulosclerosis’. From the 72 miRNAs listed, 15 were selected based on a PubMed literature review. Subsequently, mirPath v3 was utilized to identify common pathways involving these miRNAs, revealing 64 pathways with participation of at least 9 miRNAs. Key pathways like TGF-beta signaling, mTOR signaling, and Wnt signaling, known to be integral in renal disease progression, were filtered [15]. This filtration process narrowed the focus to 12 miRNAs (hsa-mir-10a, hsa-mir-21, hsa-mir-30d, hsa-mir-30a, hsa-mir-125b, hsa-mir-130b, hsa-mir-155, hsa-mir-186, hsa-mir-192, hsa-mir-193a, hsa-mir-196a, hsa-mir-200a). The top 5 miRNAs (hsa-mir-21, hsa-mir-30a, hsa-mir-193a, hsa-mir-196a, hsa-mir-200a) were selected for further analysis.

### Sample collection and urine exosome isolation

Urine samples exceeding 5 mL were collected from both patients and healthy individuals. Exosome isolation from these samples was conducted using the Total Exosome Isolation Reagent (from urine) by Invitrogen, following the provided kit procedure (Invitrogen, 2020, USA). Total RNA isolation was performed using the mirVana miRNA Isolation Kit (Invitrogen, 2020, USA). Due to low yields from miRNA-enriched isolation, we opted for Total RNA isolation. For PCR, the TaqMan^®^ MicroRNA Reverse Transcription Kit and TaqMan^®^ Small RNA Assay (Applied Biosystems, 2020, USA) were employed, with primers specific to each miRNA. The concentration of RNA isolated from the previous step was adjusted to 2 ng/μl for all samples. Since 5 μL of RNA was added into the reaction, the final concentration was 10 ng per reaction which is suitable for the process. qPCR amplification utilized TaqMan^®^ Universal PCR Master Mix II and TaqMan^®^ Small RNA Assay (Applied Biosystems, 2020, USA). All reactions were studied in duplicate.

### RT-PCR

The isolated miRNAs (hsa-mir-21, hsa-mir-30a, hsa-mir-193a, hsa-mir-196a, hsa-mir-200a) from urinary exosomes were profiled using the Applied Biosystems StepOne^™^ (Applied Biosystems, 2020, USA) in both patient and control groups. TaqMan miRNA Assays were used alongside U6 snRNA (Applied Biosystems, 2020, USA) as an endogenous control.

Relative quantification of miRNA expression was calculated using the 2^-ΔCt and 2^-ΔΔCt methods to measure fold differences between a target miRNA and a reference (ΔCt) in a particular state (e.g., disease) compared to control (ΔΔCt). The Ct is derived from a log-linear plot of the baseline-corrected PCR signal against the cycle number [16, 17]. The "fold change" is expressed as 2^-ΔΔCt.

### Statistical analysis

The association between miRNA expression levels and clinical phenotypes was evaluated using non-parametric tests, including the Mann-Whitney U test for two groups and the Kruskal-Wallis H test for three or more groups. Analyses were conducted using Python version 3.9.6, with statistical procedures carried out through the SciPy library (version 1.10.1) and data management facilitated by the pandas library (version 2.0.3). A p-value <0.05 was deemed statistically significant.

## Results

### Demographics and sample characteristics

Thirteen DKD patients, 11 FSGS patients, and 14 healthy controls were included in this study. Demographic, clinical, and laboratory findings of the patients are shown in Table 1.

**Table 1:** Demographic, clinical, and laboratory findings of the patients.

Variables	DKD group (n = 13)	FSGS group (n = 11)	Controls (n = 14)
**Age (years)**	55.8±7.4	53.0±7.2	46.2± 5.4
**Gender, male**	9 (69.2)	5 (45.4)	6 (42.0)
**Time since diagnosis (years)**	15.4± 6.0	4.5±4.9	N/A
**Creatinine (mg/dL)**	1.28±0.56	1.3 ±0.35	0.76±0.19
**eGFR (mL/min/1.73 m** ^**2**^ **)**	70.6±28.4	63.0±26.0	103.7±13.6
**Albuminuria (mg/24 h)**	1591.5±1053.3	3742.3±3061.8	*
**Proteinuria (mg/24 h)**	2349.5±1519.2	4366.9±3223.2	*

eGFR: estimated glomerular filtration rate; DKD: Diabetic Kidney Disease; FSGS: Focal Segmental Glomerulosclerosis.

Data are expressed as mean±SD for quantitative parameters and n (%) for nominal parameters.

* No proteinuria was detected in spot urine.

**Fig 1:**
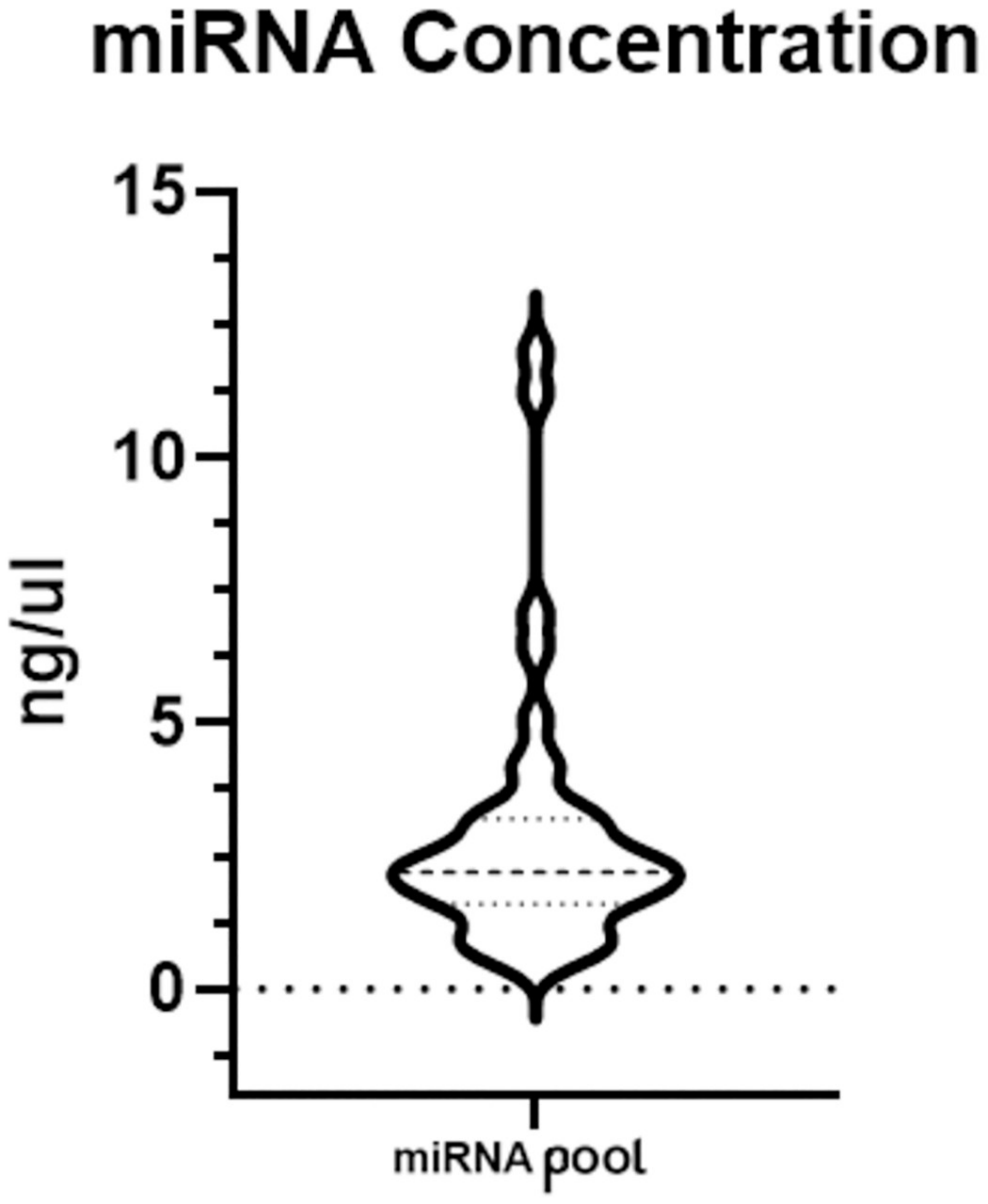
Distribution of isolated total RNA concentrations from urine samples.

For the RT-PCR step, a range of 1–10 ng of total RNA per 15 μl reaction was utilized. The distribution of total RNA concentrations isolated from urinary exosomes is given in Fig 1.

The samples were kept at -20C over approximately 2 weeks. The comparison of the U6 values measured again at the end of the study with the previously measured values is shown in Table 2. The time-dependent change of the measured Ct values is between 2–4 cycles. The results indicate that there was a molecular level loss in miRNA RT products stored for two weeks at -20C.

**Table 2:** U6 levels were measured two weeks apart (Series1-Series2). The min, max, and mean values in each individual and group are given.

	DKD (n = 13)			FSGS (n = 11)			Control (n = 14)		
Cycle Threshold (Ct)	Min.	Max.	Mean	Min.	Max.	Mean	Min.	Max.	Mean
**Series1**	24.83	30.33	27.55	25.51	33.10	28.34	25.24	30.47	28.31
**Series2**	26.91	32.00	29.92	28.84	35.70	31.95	29.26	35.06	31.69

DKD: Diabetic Kidney Disease; FSGS: Focal Segmental Glomerulosclerosis.

### miRNA analysis

hsa-mir-21 and hsa-mir-30a both displayed upregulation in the FSGS group and downregulation in the DKD group compared to the control group. For hsa-mir-21, the DKD group showed a fold change of 0.668 ± 0.25 (SE) with a highly significant p-value (p < 0.0005), and the FSGS group showed a fold change of 2.267 ± 1.138 (SE) with statistical significance (p < 0.0077). hsa-mir-30a was downregulated in the DKD group with a fold change of 0.874 ± 0.254 (SE), not reaching statistical significance (p = 0.079), while the FSGS group showed a significant upregulation with a fold change of 1.378 ± 0.312 (SE) (p < 0.0006).

The hsa-mir-193a levels showed no significant dysregulation in the DKD group, with a fold change of 1.017 ± 0.413 (SE) and a statistically significant p-value (p < 0.029), whereas the FSGS group exhibited a marked upregulation with a fold change of 4.18 ± 1.528 (SE), though this was not statistically significant (p = 0.058).

**Fig 2:**
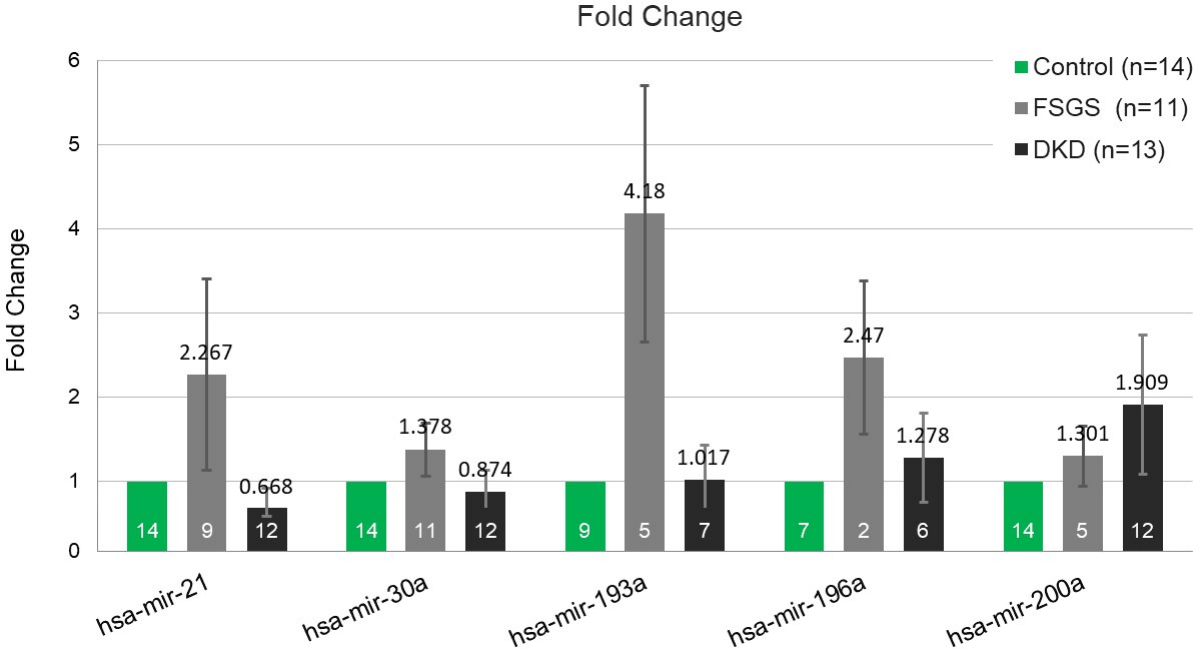
Fold changes are shown for each miRNA in DKD, FSGS, and healthy control groups.

The hsa-mir-196a and hsa-mir-200a levels were found to be upregulated in the patient groups compared to the healthy control group. For hsa-mir-196a, the DKD group showed an increase with a fold change of 1.278 ± 0.527 (SE), but the change was not statistically significant (p = 0.074). In contrast, the FSGS group exhibited a significant upregulation with a fold change of 2.47 ± 0.911 (SE) and a highly significant p-value (p < 0.0003). hsa-mir-200a was also upregulated in the DKD group with a fold change of 1.909 ± 0.825 (SE), but this was not statistically significant (p = 0.082); however, in the FSGS group, there was a statistically significant increase with a fold change of 1.301 ± 0.358 (SE) (p < 0.008). Data are shown in Fig 2 and Table 3.

**Table 3:** Fold change values in DKD and FSGS groups for each miRNA (Values calculated with ΔΔCt method).

miRNA	DKD (Fold Change) (n = 13)	Std Err	p-value	Sample size	FSGS (Fold Change) (n = 11)	Std Err	p-value	Sample size
hsa-mir-21	0.668	0.25	0.0005	12	2.267	1.138	0.0077	9
hsa-mir-30a	0.874	0.254	0.079	12	1.378	0.312	0.0006	11
hsa-mir-193a	1.017	0.413	0.029	7	4.180	1.528	0.058	5
hsa-mir-196a	1.278	0.527	0.074	6	2.470	0.911	0.0003	2
hsa-mir-200a	1.909	0.825	0.082	12	1.301	0.358	0.008	5

DKD: Diabetic Kidney Disease; FSGS: Focal Segmental Glomerulosclerosis.

## Discussion

miRNAs have garnered substantial attention as potential biomarkers for various diseases, including renal disorders like DKD and FSGS. In this study, we aimed to identify specific miRNAs that could serve as diagnostic biomarkers for these renal conditions. However, it’s crucial to acknowledge that urinary miRNA studies are highly contingent on sample conditions. The yield of exosomal miRNA from urine, which can be a diluted medium, is typically low. With advancements in biological technologies and experimental methods, miRNA screening is now feasible with minimal quantities (less than 1 ng/μl). As supported by our U6 measurements (Table 2), factors such as storage conditions and time significantly impact miRNA measurements.

In normal human kidneys, miR-193a is predominantly expressed in parietal epithelial cells [18]. Prior research has demonstrated that miR-193a targets Wilms’ tumor protein (WT1) and downregulates it [19, 20]. WT1 is a key transcription factor and regulator of podocyte development and homeostasis. Elevated levels of miR-193a in transgenic mice have been shown to induce FSGS, characterized by substantial breakdown of podocyte foot processes. Additionally, reduced WT1 expression leads to the downregulation of PODXL (podocalyxin) and NPHS1 (nephrin), among other genes crucial to podocyte morphogenesis, culminating in the collapse of the podocyte-stabilizing mechanism [19]. Conversely, in vitro studies suggest that the downregulation of miR-193a can induce the trans-differentiation of glomerular parietal epithelial cells into a podocyte phenotype, potentially aiding in the resolution of glomerular injury following acute podocyte depletion [21]. Our results indicate that in the FSGS group, even though there is a noticeable increase in hsa-mir-193a levels, the lack of statistical significance suggests caution in drawing firm conclusions. On the other hand, the findings suggest that there is a statistically significant difference in hsa-mir-193a levels in the DKD group, even though the fold change is relatively small. In this case, the statistical significance suggests that the observed change in hsa-mir-193a levels in the DKD group may be likely relevant and not just a random fluctuation. Further studies with larger sample sizes are needed to confirm or refute the observed trends in the FSGS and DKD groups.

miR-21, miR-200a, and miR-30a are associated with transforming growth factor-β1 (TGF-β1) and its downstream pathways. TGF-β1 regulates microRNAs that mediate renal fibrosis by activating Smad3 [22]. In our study, we observed increased levels of miR-30a and miR-200a in the FSGS group. The results suggest a potential involvement of miR-30a and miR-200a in the TGF-β1-mediated pathways associated with renal fibrosis in FSGS. The lack of a statistically significant upregulation of hsa-mir-200a in the DKD group, while there is a numerical increase, could be influenced by factors such as sample size, variability within the group, or other confounding factors.

TGF-β1 stimulated miR-21 targets Phosphatase and Tensin Homolog (PTEN), a tumor suppressor gene. Downregulated PTEN expression leads to increased Protein kinase B (AKT) activity, which in turn contributes to renal fibrosis in the context of mesangial cell hypertrophy and increased matrix protein synthesis [23]. Additionally, miR-21 downregulates Smad7, creating a feedback loop that inhibits miR-21 activity itself [24]. Our findings of upregulated miR-21 levels in the FSGS group could support its role in fibrosis. However, the slightly reduced levels in the DKD group warrant further investigation to understand this differential expression.

Another focus is miR-30a, which is downregulated by the TGF-β downstream pathway. This miRNA targets DNA Methyltransferase (DNMT) 1 and DNMT3a. Reduced miR-30a levels can lead to increased DNMT, promoting the methylation of the Klotho promoter and reducing the activity of the Klotho protein. The reduction in Klotho activity is associated with the potentiation of renal fibrosis [25]. Our observation indicates the differential regulation of miR-30a in DKD and FSGS suggests distinct molecular mechanisms underlying these kidney disorders. The upregulation of miR-30a in FSGS may indicate a potential protective or compensatory response, whereas the downregulation in DKD, although not significantly, may contribute to disease progression.

In podocytes, the expression of TGF-β1, thrombospondin-1 (TSP-1), TGF-β type II receptor (TGF-β2R), and phosphorylated Smad2-Smad3 is reported to be substantially increased in FSGS [26]. miR-196a and miR-196b are predominantly expressed in both glomerular and tubular interstitial cells and target TGF-β2R. miR-196a and miR-196b have been demonstrated to have an inhibitory role in the progression of renal fibrosis through downregulation of TGF-β2R in the mouse renal fibrosis model [27]. The observed rise in urinary miR-196a levels in our study may indicate a response to elevated TGF-β1 levels, notably in the FSGS group.

This study has several limitations that need to be addressed. First, the small sample size could potentially increase the likelihood of false-positive findings and diminish the statistical power of the results, particularly in miRNA studies. To achieve more reliable and robust results, it is essential to increase the sample size in future studies. Second, the study was conducted in a limited geographic area, the results might not be generalizable to a broader population. Third, the study is cross-sectional without longitudinal follow-up, it may limit the understanding of miRNA levels over time in disease progression. Finally, analyzing circulating miRNA for disease markers poses challenges in achieving high accuracy. Pre-analytical variables like sample selection, storage, preparation, and extraction introduce uncertainty. Despite controlled handling, the risk of false results persists due to normalization issues and potential statistical misinterpretation.

In conclusion, our preliminary findings indicate that certain miRNAs, such as miR-21, miR-30a, miR-196a, and miR-200a, exhibit elevated expression levels in the FSGS group, while miR-21 levels are reduced in the DKD group. These specific miRNA expression patterns suggest their potential involvement in the pathogenesis of kidney diseases. Additionally, these miRNAs hold promise as potential biomarkers for discerning between patients with FSGS and those with DKD. However, the limited sample size in our study necessitates further investigation to differentiate more accurately between these disease groups.
